# Ethnocultural empathy development of future language teachers through digital multiliteracy resources for low-literacy adult migrants

**DOI:** 10.3389/fpsyg.2024.1398457

**Published:** 2024-06-05

**Authors:** Analí Fernández-Corbacho, Esther Cores-Bilbao, Patricia Flor-Arasil

**Affiliations:** ^1^English Studies Department, Universidad de Huelva, Huelva, Spain; ^2^Faculty of Humanities and Social Sciences, Universidad Isabel I, Burgos, Spain; ^3^Faculty of Health Sciences, Universidad Internacional de Valencia, Valencia, Spain

**Keywords:** ethnocultural empathy, language teacher education, critical thinking skills, multiliteracy, digital resources, multimodality

## Abstract

The increasingly digital and multicultural 21st-century society requires future teachers to be prepared for the changes and challenges they may encounter. Not only language and digital competences, but critical-thinking and problem-solving skills are needed. Moreover, well developed socio-affective abilities, empathy among them, are also key when dealing with others. This is even more relevant when teachers are to work with a non-mainstream population, such as adult migrants with low literacy levels, and to design student-centered curricula or activities. Empathy is a multifaceted process involving, among others, perception, intellection, affect and other sensory aspects of the lived experience. It has been argued that the first-person perspective-taking involved in empathic engagement must necessarily involve rational computation and cognitively mediated processing. Training future teachers in the Pedagogy of Multiliteracies is a means to integrate multimodal digital instruction and aggregate cognitive as well as socio-emotional features to the education of future language teachers.

**Method:**

A mixed-method pre-post study was conducted with 48 trainee teachers who participated in stand-alone digital multiliteracy interventions, in which they were encouraged to envisage themselves as future teachers of low-literate migrants. Policy documents such as the reference guide on Literacy and Second Language Learning for the Linguistic Integration of Adult Migrants, journal articles, audiovisual resources as well as examples of existing educational materials aimed at the target audience, were made available to them on an online platform. In two separate studies, trainees were encouraged to collaboratively produce two different multimodal outputs. The Revised Scale of Ethnocultural Empathy was administered before and after the intervention, subjecting the data obtained to quantitative analysis. Qualitative data was also collected to gain a better understanding of the affective and cognitive processes experienced by the participants.

**Results:**

Simple statistical analysis coupled with the comparison of means was used to respond to the research questions. Statistical hypothesis testing, including correlations and non-parametric statistics were used to analyze the relationship between each of the factors within the RSEE and the participants, considering the different interventions applied. Non-parametric tests (U-Mann Whitney) were used to compare the differences between the levels of ethnocultural empathy of the participants in the two studies. Significant differences were found in Factor 3 (Empathy) and Factor 5 (Anxiety) between the groups and their post-intervention results, with a *p* value of 0.053 and 0.038, respectively. The effect size r was calculated, obtaining a size effect of 0.625 for Factor 3 (Empathy) and 0.674 for Factor 5 (Anxiety). These results indicate that the significant differences and the size effect between both groups are large. U-Mann Whitney non-parametric analysis also revealed gender differences in Factor 3 (Empathy), showing females higher levels than males. Effect size r analysis showed a large size effect of 0.708 for Factor 3 (Empathy). The findings pertaining to gender-related differences in empathy levels confirm the conclusions drawn by previous studies. When contrasting study 1 and 2, statistical differences were also shown after the intervention for the ‘Anxiety and Lack of Multicultural Self-efficacy’ factor. The qualitative data analysis was carried out with Atlas.ti v.8, in order to isolate and categorize the broader themes and the most significant explanatory quotes extracted from the participants’ records and interviews. The results reveal the learning strategies that each group of learners applied to successfully complete the task at hand, as well as the participants’ deployment of their critical thinking skills and the awakening of a sense of awareness of their own professional competence development process.

**Conclusion:**

This study set out to compare how effective two digital multiliteracy interventions were in developing future language teachers’ ethnocultural empathy and cognitive abilities when appraising the educational needs of low-literacy migrants. Despite the small sample size, the study certainly adds to our understanding of the impact of multimodal tasks involving critical thinking skills on trainees’ cognitive and affective abilities. Besides, it expands the growing body of research that points to the desirability of embedding digitally-based content creation tasks in training curricula for future language teachers.

## 1 Introduction

In an increasingly diverse society, with constant migration bringing people from different linguistic and cultural backgrounds into the educational systems of receiving countries, teachers need to be well prepared for the changes they will encounter. In particular, language teachers play a critical role in facilitating the integration of migrants into their host communities (Carlsen et al., 2023), as becoming literate in an additional language is often part of the survival kit that migrants need. In fact, individuals whose language is different from the dominant one in the country tend to have lower literacy levels, a tendency increasing with age (OECD, 2017). This also reduces migrants’ employability rates, wages (Himmler and Jäckle, 2018), in general, hinders their successful participation in society (Joyce, 2019), and potentially leads to social exclusion (Leone et al., 2005). This situation is replicated from one generation to the next, as children of parents with low educational attainment and a lower socio-economic status are prone to have poor literacy skills themselves (Hemmerechts et al., 2017; Fernández-Corbacho, 2018). The circumstances are even more complex for those migrants who, upon arrival, are faced with the need to acquire both oral and literacy skills in the target language, which poses additional problems for communication and involvement in the societies they wish to live in (Minuz et al., 2022). They typically find themselves in a situation where they have little exposure to the new language, lack the time to attend classes due to family and work obligations; and, if able to attend class, the large variance in what are often mixed-ability classrooms prevents individuals from receiving the attention they need to progress (Haznedar et al., 2018). Consequently, language teachers should not make assumptions about their learners’ abilities and should be aware of the diverse and complex cognitive skills they need to develop (Gonzalves, 2021), as they are different from the mainstream learners they are usually trained to attend to. Namely, adult migrants are already speakers or readers of other languages and these home languages are valuable resources that can help them become interculturally competent citizens, as Haznedar et al. (2018) point out. Moreover, being orally competent or literate in the home language reduces marginalization and increases empowerment within the community. In all cases, these adults require qualified teachers who provide the specialized instruction necessary to reflect the diverse language profiles of individuals in a classroom (Haznedar et al., 2018).

Regarding educational contexts, migrant students are reported to have decreased levels of *affective happiness* (Rodríguez et al., 2020). Furthermore, research has shown that the negative stereotypes that educators hold about migrant pupils are detrimental to how they perform academically (Froehlich et al., 2022). Thus, language teachers need to gain an understanding of the affective factors that might have a positive or negative bearing on learners’ performance, such as beliefs, identity, anxiety or motivation, which are particularly useful when considering the adult language learning process (Fonseca-Mora, 2023). However, Canales and Rovira (2016) report the scarce development of socioemotional or socio-personal competencies in teacher training, attributing it to the current lack of emotional literacy in society, and insisting on the need to address psychosocial well-being and empathy in teacher training courses. It is also of utmost importance that teachers be aware of their own bias towards diversity in their classroom; in this sense, fostering teacher empathy is key. It is therefore imperative to address this concern in teacher education programs, which must be able to respond to the needs of adult learners with a migration background, arising from the unique experiences and challenges they face in their daily lives. Adult migrant learners may be exposed to situations of violence, racism and hostility that affect their ability to participate fully in language learning (Baynham, 2006). With these potential struggles in mind, Mercer (2016) outlines how promoting empathy improves group dynamics and cultivates cooperative and collaborative relationships among students, which, in turn, fosters an environment conducive to effective language learning and the strengthening of interpersonal and communicative skills.

Despite these earlier contributions, a recent review of trainee teachers’ practical experiences in their classroom interactions reveals that student teachers often harbor negative beliefs and concerns about working with learners of non-native backgrounds, while acknowledging the importance of empathy in their training to educate them (Hanna, 2023). Cognitive perspective-taking, implying the integration of one’s own and others’ mental representations, has been found to mitigate intergroup assumptions, biases and preconceptions towards stereotypical target groups, and has therefore been proposed as a possible means of stimulating the willingness to engage in cross-group contact and initiate rapprochement-oriented actions (Wang et al., 2014). Mutual comprehension towards others and readiness for intergroup collaboration might also be attained through fostering empathy, that is, the affective state elicited by experiencing the emotions of the other, in the awareness that those third-party emotions are the source of one’s affective state (de Vignemont and Singer, 2006). This cognitive-affective dichotomy may be problematic, as earlier works have identified how perspective-taking towards oneself may attenuate positive emotions (Wallace-Hadrill and Kamboj, 2016) or even exacerbate out-group rejection (Klimecki et al., 2020).

Given that cultivating positive other-regarding emotions is thought to be one of the mechanisms for inducing empathy towards individual subjects, which can generalize to favorable attitudes towards other members of the target group (Klimecki, 2019) and which is essential for effective intercultural communication (Mercer, 2016), further research exploring these links has been encouraged. In this light, Martínez-Otero Pérez (2011) raises the need to promote empathy in university curricula for the training of education professionals, placing special emphasis on the incorporation of the empathic intersubjective educational style in the instructional process. Such style, which entails a “balanced cognitive and affective approach to the emotional reality of others” (equilibrada aproximación cognitiva y afectiva a la realidad emocional ajena) (Martínez-Otero Pérez, 2011, p. 187), is instrumental in the training for future teachers and educators. Similarly, Warren (2018) argues for the provision of a curriculum that supports empathic dispositions in teacher education programs as a forerunner of culturally responsive teaching approaches which cater to culturally and linguistically diverse students. Thus, language classes for migrants should be adapted to enable effective learning of a new language in a context that recognizes and respects their cultural identities and prior experiences, so that “the identity of ‘student’ itself can constitute a stable point in the highly unstable lifeworld” (Baynham, 2006, p. 25).

Whilst general empathy may be defined as the ability to relate to another person’s emotions with a certain degree of understanding of their emotional state (Decety and Jackson, 2004), ethnocultural empathy, also referred to as cross-group empathy (DeTurk, 2001), could be defined as the empathy felt towards people from other cultures which, in order to thrive, challenges obstacles not encountered in having empathy towards those belonging to the same cultural community. In that vein, the first instrument available for assessing ethnocultural empathy, the Scale of Ethnocultural Empathy (Wang et al., 2003), adopted a culture-oriented framework with and ethnic perspective, and operationalized ethnocultural empathy as composed of three dimensions: intellectual empathy, that is, “ability to understand a racially or ethnically different person’s thinking and/or feeling”; empathic emotions, which implies “to the feeling of a person or persons from another ethnocultural group”; and communicative empathy, which is the “expression of ethnocultural empathic thoughts (intellectual empathy) and feelings (empathic emotions)” (p. 222). Nonetheless, Rasoal et al. (2011b) found “strong correlation between *basic* and ethnocultural empathy” (p. 927), implying that the two overlap considerably. In this regard, both are regarded as flexible human capacities, whereby basic empathy is “susceptible to social-cognitive intervention, such as through training or enhancement programs for targeting various goals” (Decety and Jackson, 2004, p. 94) and ethnocultural empathy is deemed “something dynamic that can be learned and developed over time” (Rasoal et al., 2011a, p. 8), and therefore, amenable to expansion. In the same vein, Meskill (2005) found that in-service and pre-service foreign language teachers gained greater sensitivity to intercultural differences when teacher education programs featured training opportunities to foster their empathy towards students from other countries.

More recently, a range of studies have underscored the importance of intercultural empathy for pre-service second language teachers. Whitford and Emerson (2019) found that cultural empathy positively correlates with multicultural sensitivity and understanding and can thus reduce implicit out-group bias. Kapıkıran (2023, p. 11510) affirms that ethnocultural empathy may reduce “conflicts and discriminatory attitudes in preservice teachers.” Trainee teachers’ reported levels of ethnocultural empathy score high (Tutkun, 2019), being attributed to the fact that teaching is strongly correlated with altruistic behavior and empathetic attitudes. In other words, second language teachers are ideally placed to mediate the successful socialization of migrant learners in the local school setting, as they tend to show higher levels of openness to new situations, empathy and understanding of the difficulties faced by multilingual learners (Rokita-Jaśkow, 2023). Indeed, when working with a diverse student population, the appropriate use of “intercultural empathy and positive affect ensures effective communication” (Yang et al., 2019, p. 309) and supports the building of rapport between diverse students and teachers. Along the same lines, Hanna (2023) posits that disregarding the role of empathy as a core value for prospective teachers is linked to the difficulties in nurturing critical self-reflection among aspiring educators and raises questions about the adequacy of initial teacher education in preparing them for engaging effectively with migrant students.

As Mercer (2016) points out, trainee teachers’ awareness of the importance of interpersonal skills in language learning can help them to assess the potential effects that such skills have on classroom life and cross-cultural communication. Prospective empathetic teachers therefore require cognitive skills to interpret emotional cues, adopt diverse perspectives, and foster an inclusive and supportive learning environment (Underhill, 2013). In addition, critical thinking (CT) skills remain essential in equipping qualified citizens to face the challenges of ever-shifting digital environments that are transforming society (Lincoln and Kearney, 2019). Along the same lines, Mallinckrodt et al. (2014) point to the expanding body of research linking multicultural university programs to increases in students’ empathy, intergroup understanding, communication skills and CT skills. CT is thus even more central to teacher education, since learners need to be provided with the skills to become effective and creative educators of new generations. Research shows that higher levels of teachers’ cognitive skills raise pupil performance significantly (Hanushek et al., 2014). However, teachers first need to develop CT themselves to promote it in their students (Lorencová et al., 2019).

Over the past few decades, the concept of CT has been iteratively addressed and defined. However, the conceptual framework developed by the Delphi Committee in 1990 is probably the most widely used in research on the subject. The so-called *Delphi Report* defines CT as “purposeful, self-regulatory judgment which results in interpretation, analysis, evaluation, and inference, as well as explanation of the evidential, conceptual, methodological, criteriological, or contextual considerations upon which that judgment is based” (Facione, 1990, p. 3). Apart from this definition, six key cognitive skills, which the ideal critical thinker is expected to develop, have been identified: interpretation, analysis, evaluation, inference, explanation and self-regulation, each of them comprising several sub-skills (Table 1).

**Table 1:** Framework of critical thinking skills (Facione, 1990, 2020).

**Skills**	**Sub-skills**
1 Interpretation	Categorization
	Decoding significance
	Clarifying meaning
2 Analysis	Examining Ideas
	Identifying arguments
	Analyzing arguments
3 Evaluation	Assessing claims
	Assessing arguments
4 Inference	Querying evidence
	Conjecturing alternatives
	Drawing conclusions
4 Explanation	Stating results
	Justifying procedures
	Presenting arguments
5 Self-regulation	Self-examination
	Self-correction

Although research points out that effective CT development requires explicit instruction for learners (Abrami et al., 2008; Marin and Halpern, 2011), some positive results can also be achieved, albeit to a lesser extent, without working specifically on CT (Lorencová et al., 2019). However, other variables can affect the effectiveness of such interventions, like teachers’ specific training on CT (Lorencová et al., 2019). In any case, as Dwyer et al. (2014) point out, the use of CT skills should be encouraged in all problem-solving and other real-life tasks undertaken in the classroom, as learners need to be prepared to critically appraise new information “so that they can constructively solve problems, draw reasonable conclusions and make informed decisions” (p. 50). Not only is teaching CT crucial, as it develops decision-making and problem-solving skills, but it also supports the development of dispositional factors (Dwyer et al., 2014) that lead to acting critically. That is, it is a two-way relationship, in which both CT and higher-order thinking skills come into play when learners have to make decisions to complete a complex task that requires effective problem-solving (Snyder and Snyder, 2008). Thus, CT skills could be developed through the introduction of action-oriented tasks whereby learners are expected to collaborate and mediate in producing a multimodal final product which also involves problem-solving skills.

In our ever-changing digital and multicultural societies, developing literacy skills among migrant populations can be a challenging task for educators. In fact, the concept of literacy has evolved over the last decades, not only referring to reading and writing, but extending to the OECD concept of “understanding, using, evaluating, reflecting on and engaging with texts in order to achieve one’s goals, to develop one’s knowledge and potential and to participate in society” (2019, p. 28). This definition also includes screen-based text, which is in line with current common digital practices. Indeed, the texts that we use to communicate contain multimodal elements, such as visual, oral, or gestural input, reflecting the ways in which people make meaning depending on their sociolinguistic and cultural backgrounds. Those enriching socially-situated literacy practices are acknowledged by the Pedagogy of Multiliteracies (New London Group, 1996) and have an impact on literacy development (Cope and Kalantzis, 2000). In this pedagogical approach learners are considered active participants, producers of texts that convey the diversity of our multilingual digital society. In the classroom, this requires inclusive and equitable pedagogical practices (Warren and Ward, 2019) that spur a process of transformation in learners. Consequently, the multiliteracy-based Learning by Design framework (Yelland et al., 2008) puts an emphasis on teaching practices that require learners’ deep reflection and decision making. In this line, previous studies advocate for the use of multiliteracies to develop learners’ CT skills. Zhang-Leimbigler (2014) affirms that when a multiliteracies approach is introduced, students become more involved in observing and analyzing their performance, and in transferring their learning to new situations. In fact, critical literacy is central to multiliteracies, as Warner and Dupuy (2018) assert, “literacy involves critical thinking and in particular the awareness that texts are not neutral” (p. 124). On the other hand, critical literacy requires educators to be active designers of social change. That is, multiliteracies call for literacy practices that lead to social justice through a critical approach to learning in language teacher education programs, which should promote the recognition of diverse meaning-making practices. Thus, when working with diverse learners, multiliteracies create spaces for analyzing the complex situations and experiences of adult learners. In the case of migrant learners, well-trained educators can help them to transfer their skills from familiar contexts to the new situations they encounter in the host countries.

As indicated above, the concept of multimodality is central to multiliteracies: a multimodal way of meaning making includes linguistic (oral and written), visual, audio, gestural, tactile, and spatial patterns (New London Group, 1996). Thus, individuals have to learn how to acquire and construct knowledge from many different sources and representation modes. In fact, previous research (Gouthro and Holloway, 2013) has found benefits for culturally and linguistically diverse learners when they use more inclusive broader literacy practices, associated with multiliteracies. A multimodal approach to literacy can promote lifelong learning; multiliteracies also promote a positive approach to language diversity, and a more flexible and multilingual approach to language learning, for example through the recognition and validation of the use of vocabulary from learners’ mother tongue (Holloway and Gouthro, 2020). As stated in Ávila-López and Rubio-Alcalá (2023) systematic review, effective multiliteracies teaching practices embrace the potential of multimodalities to improve multilingual competences. Moreover, “multiliteracies and multimodality foster creativity and criticality, engage marginalized learners, and provide greater versatility in meaning-making practices” (Holloway and Qaisi, 2022, p. 85). Consequently, taking a multiliteracies approach to language teaching requires educators to acknowledge the different modalities in which students engage in the learning process (Holloway and Gouthro, 2020).

As García-Barroso and Fonseca-Mora (2023) conclude in their narrative review, the use of digital technologies facilitates literacy development of adult learners of additional languages by promoting the digital practices and skills required for navigating the multimodal texts encountered in everyday life. In fact, digital technologies are not new to migrants, who, in many cases, are defined as “connected migrants,” based on the digital communication practices during their migratory experience (Diminescu, 2008). Furthermore, they engage in multilingual practices that promote their literacy skills (D’Agostino and Mocciaro, 2021). Regarding educators, it has been argued that the use of digital tools and adapted computer and authoring technologies raises trainee teachers’ interest in their future career and in the development of a culture of empathetic behavior to a greater extent than the application of traditional training methods (Salamatina, 2019). Similarly, previous studies have shown that the use of audiovisual material in the training of future language teachers can cultivate empathetic attitudes, enabling trainees to relate to their future learners and appreciate the challenges that those learners might experience during the L2 acquisition process (Lee, 2019). New avenues of research have recently been pursued within this domain, extending the existing traditional notion of empathy to the digital realm and conceptualizing constructs such as *digital empathy*, understood as “the cognitive and emotional capacity to be reflective and socially responsible in the strategic use of digital media” (Jiang and Gao, 2020, p. 72). Fundamentally, Jiang and Gao’s study (Jiang and Gao, 2020) sought to identify empathic changes, as well as their affective or cognitive nature, arising from an innovative pedagogical intervention which engages foreign language learners in the creation of multimodal content. One conclusion that emerges is the need for teachers and practitioners to embrace a multimodal approach to language teaching, regarding language as a semiotic device for meaning-making and broadening the focus of language learning to achieve “more empathetic and responsible literacy practices through digital tools” (Jiang and Gao, 2020, p. 83). Such practices enhance teacher education by helping student teachers to become critically reflective educators.

In the light of the studies reviewed above, the undertaking of action-oriented tasks that require learners to collaborate and mediate concepts and communication in order to make decisions, solve problems and produce a multimodal product aimed at low-literacy migrants could promote future teachers’ ethnocultural empathy and CT skills. Thus, in our study, the main objective of the interventions carried out was to raise learners’ awareness of the linguistic and social needs of adult migrants for their inclusion into the host society. In addition, learners’ empathy toward the migrant population was expected to be developed. Besides, by introducing multiliteracy activities focused on the development of empathy, we expected learners to activate self-regulating CT skills. To that end, this study set out to compare how effective two digital multiliteracy interventions were in developing future language teachers’ ethnocultural empathy and cognitive abilities when appraising the educational needs of low-literacy migrants.

Specifically, the research questions guiding our study are:

RQ1: Can student teachers’ ethnocultural empathy be developed by designing multimodal products related to low literacy adult migrants?

RQ2: Does ethnocultural empathy unfold differently depending on the type of multimodal product designed by student teachers?

RQ3: What differences can we observe across the other variables studied in the sample, such as sex, CT skills or academic attainment and the development of ethnocultural empathy?

RQ4: What cognitive processes/abilities and CT skills are promoted while designing multimodal products intended for adult migrants?

RQ5: What perceptions about their experience during the production of multimodal outputs do participants report?

## 2 Methods

### 2.1 Participants

A total of 48 participants initially started the research. Their informed consent was obtained at the beginning of the study. A total of 6 participants were excluded from the study due to discontinued involvement or age disparity. They were a group of third-year undergraduate students of both English Studies and the double degree program in English and Hispanic Philology in an Andalusian university, who were enrolled in a language acquisition course featuring a language teacher education module. They were randomly split into two different groups (study 1 and study 2) depending on the task they were assigned. Therefore, the participants in study 1 were instructed to create teaching materials; whilst the participants in study 2 had to create a video. When considering the total group, the sample was not representative in terms of gender. Of the final sample of 42 participants 13 were male (31% of the total sample) and 29 were female (69% of the total sample), with mean age of 21 years old and standard deviation of 1,274; the age range was between 20 and 25 years old. In turn, the group that participated in study 1 comprised a total of 11 male participants (46%) and 13 female participants (54%); the group that participated in study 2 consisted of a total of 2 male participants (11%) and 16 female participants (89%). Table 2 summarizes the descriptive statistics for the sample.

**Table 2:** Sociodemographic descriptive statistics for the sample.

		Frequency	Percentage
Volunteering	No	38	90.5
	Yes	4	9.5
Mother tongue	Spanish	36	85.7
	French	1	2.4
	German	1	2.4
	Turkish	3	7.1
	Spanish_Darija	1	2.4
Nationality	Spanish	35	83.3
	French	1	2.4
	Turkish	2	4.8
	Moroccan	1	2.4
	Chinese	1	2.4
	Mexican	1	2.4
	Italian	1	2.4
English level	B1	4	9.5
	B2	13	31
	C1	22	52.4
	C2	3	7.1

The group is characterized by a predominance of Spanish participants (83.3%) and a small but varied representation of other nationalities (16.6%) and mother tongues. The participants were also asked about their experience in volunteering programs with migrants. Again, there is a large majority who have no experience in this context, in fact, only 9.5% had been volunteers working with the migrant population. Besides, 25% had attended a workshop where they had been interacting with migrants. However, the high variability and low representation of each group makes it difficult to carry out statistical analyses for comparative purposes.

### 2.2 Instruments

#### 2.2.1 Revised scale of ethnocultural empathy, Spanish version

Finck et al. (2021) adapted the Everyday Multicultural Competencies Scale/Revised Scale of Ethnocultural Empathy (EMC/RSEE; Mallinckrodt et al., 2014) to be administered to Spanish speaking university students in Colombia. This validated Spanish version has introduced some changes to the original tool: the number of items is slightly reduced, and cognitive and affective empathy are merged into one single factor. Thus, the 5 resulting factors are: (1) “Cultural Openness and Desire to Learn” (Cultural Openness hereafter), (2) “Awareness of Contemporary Racism” (Awareness of Racism, henceforth), (3) “Empathy,” (4) “Resentment and Cultural Dominance” (from now on, Resentment) and (5) “Anxiety and Lack of Multicultural Self-Efficacy” (hereafter Anxiety). The scale is based on the notion of intercultural competence, which comprises communication as well as “effective and appropriate behavior across intercultural situations” (Finck et al., 2021, p 164), and aims at measuring learners’ empathy toward population from diverse linguistic and sociocultural backgrounds, as well as considering issues of racism or the lack of self-efficacy in members of the dominant cultural community. All the items can be found in Finck et al. (2021, p. 175).

#### 2.2.2 Reflection logs and interview

At the end of the interventions different tools for collecting qualitative data were used. In study 1 each participant completed a reflection log. They were asked to reflect on the difficulties and positive aspects of the project, their perception of what they had learnt, what had helped them, and their opinion about the products they had designed. In study 2, a group of three students participated in an in-depth semi-structured interview. They were all asked questions about the intervention, they were encouraged to talk about the final task they had completed and give their opinion about the knowledge they had gained and how it could impact their future teaching practices.

#### 2.2.3 Final products designed by participants

Data on their academic attainment has been collected through the grades they received in the final products they designed. Students participating in study 1 were divided in groups of 4–5 and required to design teaching materials for migrants, more specifically, a bilingual (English–Spanish) teaching unit to teach the target language to low-literacy adult migrants. The materials created had to fulfil several criteria: select a relevant topic for the target learners; include the descriptors presented in the reference guide on Literacy and Second Language Learning for the Linguistic Integration of Adult Migrants (LASLLIAM; Minuz et al., 2022); identify an adequate difficulty level; and adhere to a multimodal design.

In study 2, participants divided in groups of 3 were asked to read an article about the situation of low-literacy migrants. Then, participants had to create a video presenting the key contents and the conclusions they drew from the reading. They also had to add images, subtitles, and their own voices. This ensured the delivery of a multimodal, audiovisual digital product. At the end of the video, 3 comprehension questions for viewers had to be included. Both conceptual and technical aspects were considered in the evaluation of students’ video submissions.

### 2.3 Procedures

#### 2.3.1 Intervention

The intervention took place during the second semester of the academic year 22/23, in a module specifically related to teaching languages to migrants within a language acquisition course. Two studies were carried out. In both cases, a digital technology-supported six-week intervention was delivered in a language teacher education course. The main objectives were to raise prospective teachers’ awareness of the linguistic and social needs of adult migrants, as well as to promote participants’ empathy toward the migrant population and to activate self-regulating CT skills. A wide range of resources were made available in an online platform: policy documents, such as the reference guide on Literacy and Second Language Learning for the Linguistic Integration of Adult Migrants (Minuz et al., 2022), journal articles, audiovisual resources and examples of existing educational materials intended for the target audience. Learners were encouraged to make use of interactive online tools for in-class and out-of-class activities when needed. Both interventions were similar in terms of contents and materials used, but learners were required to design two different final products. The interventions followed the same pattern: the first step focused on getting familiar with the main concepts dealt with in the project: literacy, multiliteracies or multimodality; the second step zoomed in on the target population, who they are, or their linguistic and social needs. Finally, in study 1 existing teaching materials were analyzed in terms of their appropriateness for the target group.

The main task students had to undertake was different for each of the two groups. As described above, in study 1, learners were distributed in seven heterogeneous groups, consisting of students from both degree programs and international students. and were asked to design teaching materials. They were expected to understand the main concepts of the project, consider migrants’ needs, critically analyze teaching materials, and finally apply what they had learnt to create new teaching materials. In study 2, learners, organized in eight groups, focused on the trajectories of low-literacy migrants. After reading a text, selected for its appropriateness to the students’ knowledge and assignment objectives (see Adami, 2008), learners had to turn the written information into a digital product. To do this, they had to identify the main ideas, explain them in their own words, and illustrate them with examples. In this case, learners were expected to be able to analyze and synthesize the information in the article, to present it using their own words, accompanied by relevant pictures and other visual cues. As for technical and digital elements, they were required to include subtitles and narrate the resulting text themselves in voice-over. Both contents and technical aspects were evaluated. In both studies, the final products had to be uploaded to the online platform used in the course.

The interventions followed the guidelines of the Leaning-by-Design framework (Yelland et al., 2008), which promotes the use of problem-solving and higher-order thinking skills, and is based on the Pedagogy of Multiliteracies (New London Group, 1996; Cope and Kalantzis, 2000). In both cases, learners worked in groups to make decisions and collaborate, in order to successfully complete the task. They had to compromise to reach agreements within their groups, but also to put themselves in the shoes of adult migrants and try to understand migrants’ reality and difficulties so that they could fully grasp their educational needs (in study 1), whereas in study 2 they also had to mediate the written text and transform it into a multimodal audiovisual product.

The RSEE was administered before and after the interventions. Besides, once the interventions had finished, the participants in study 1 were asked to complete a reflection log to ponder on the difficulties encountered and positive aspects they had experienced during the project. In study 2, an in-depth semi-structured interview was conducted. Discussion was prompted by a predetermined set of questions which led to a more open exchange among the participants.

#### 2.3.2 Data collection

Quantitative data was collected via *Google Forms,* where participants had to self-generate a unique code to protect anonymity. Apart from the answers to the RSEE, the form collected demographic and other relevant information, such as age, gender, nationality, or volunteering experience. The RSEE items follow a *Likert* format with responses ranging from 1 = strongly disagree, to 6 = strongly agree. Later, the final grades obtained in the allotted task were added to the database.

Following the administration of the scale, qualitative information was collected to better understand participants’ experiences. This approach makes it possible to describe subjectively perceived phenomena at the individual and group level in greater detail and to explore the links between the themes mentioned by the participants (Mezmir, 2020). Following Mezmir’s recommendation to “ensure that a range of different cases, sources, and time periods are reviewed” (Mezmir, 2020, pp. 17–18), two different techniques were applied to obtain the qualitative data: the compilation of logs written by the students in study 1, and in-depth semi-structured interviews with three participants selected from study 2. The use of the reflection logs and the interviews is in line with previous studies that have reported results related to critical thinking “based on qualitative methods, such as an analysis of texts (i.e., reflective reports, blog entries, …)” (Lorencová et al., 2019, p. 8). Thus, the qualitative analysis of the participants’ narratives was expected to provide information about the learners’ cognitive processes and critical thinking skills, as well as other aspects related to the design of the final products. Finally, students’ grades were collected at the end of the intervention.

### 2.4 Data analysis

#### 2.4.1 Quantitative analysis

The first part of the quantitative analysis consisted in the generation of the descriptive data for all variables, including sex, age, nationality, mother tongue and volunteering experience. Later, the five factors included in the RSEE were analyzed. Factor scores were used to analyze the 5 factors of the RSEE scale. As the RSEE used was not validated for the target population in this study, using factor scores would facilitate the interpretation of the results. The computed factor scores are standardized to a mean of zero. Sex was classified as male and female. Non-parametric tests were used to compare the differences between participants for study 1 and study 2, and sex with the different factors of the RSEE. Spearman correlations and the proposal of a moderation model was intended. Statistical analysis was performed using SPSS software (version 25).

#### 2.4.2 Qualitative analysis

Data from the student logs and transcripts from the in-depth interviews were compiled and imported into the qualitative analysis software *Atlas.ti v.8* after being anonymized.

Coding, searching for themes and clustering were carried out according to the framework of CT skills and sub-skills proposed by Facione (1990, 2020), as well as to other emerging categories identified during the text analysis process. Each of the three researchers independently reviewed the data, generated the initial codes, and refined them collaboratively in a shared codebook, following consensus reached in group discussions to ensure consistent coding practices.

Data reduction was done by grouping the codes into broader categories and overarching themes. Axial coding was then undertaken using the *Atlas.ti* v.8 functionalities to remove overlapping codes and redundancies, revealing the dominant codes, matching them with their sub-categories, and determining the attributes and dimensions of each of them. Finally, the quotations extracted from participants’ logs and interviews were assigned the corresponding codes.

**Figure 1:**
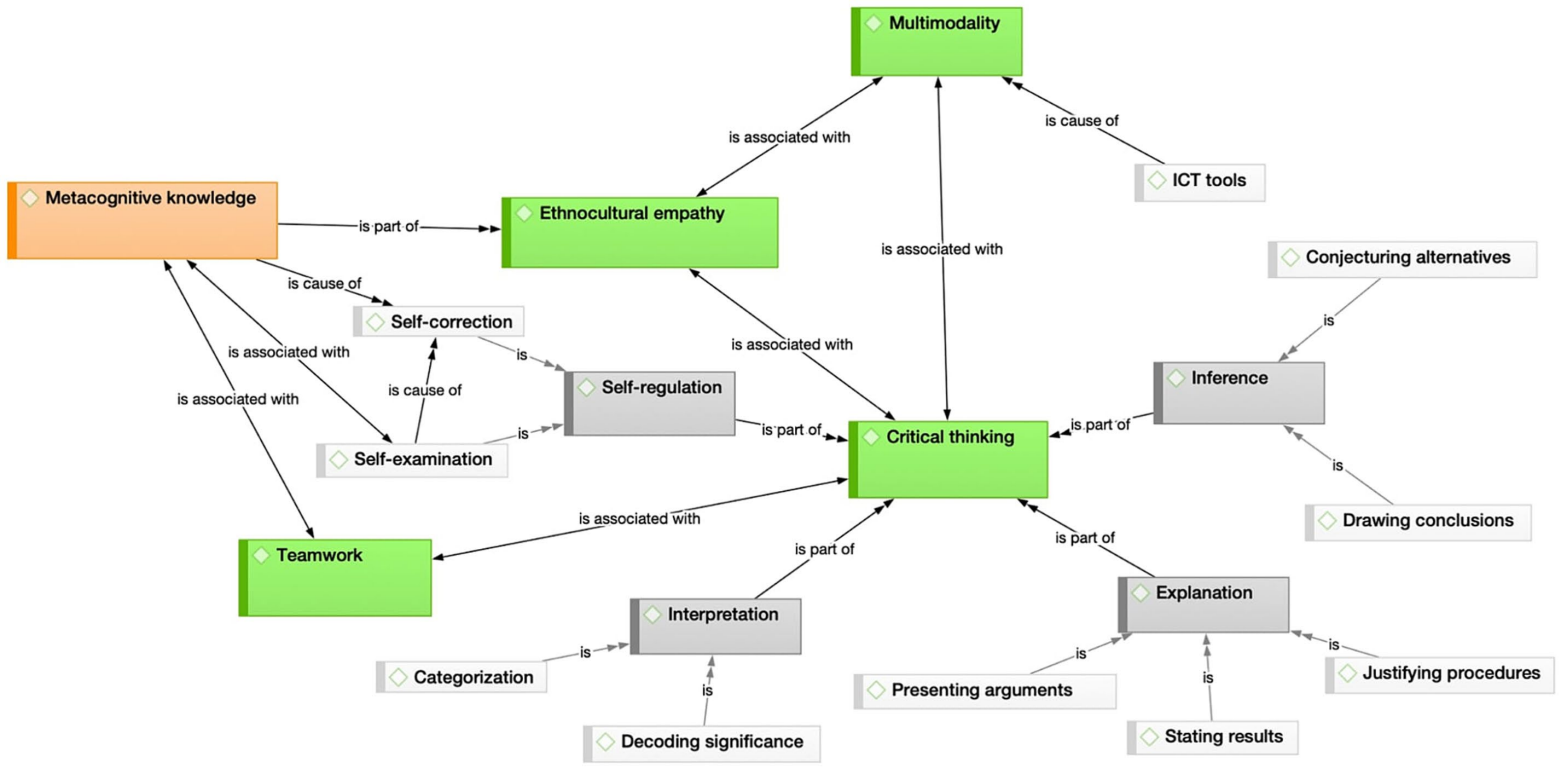
Relational patterns between the topics that emerge in the students’ logs (study 1).

**Figure 2:**
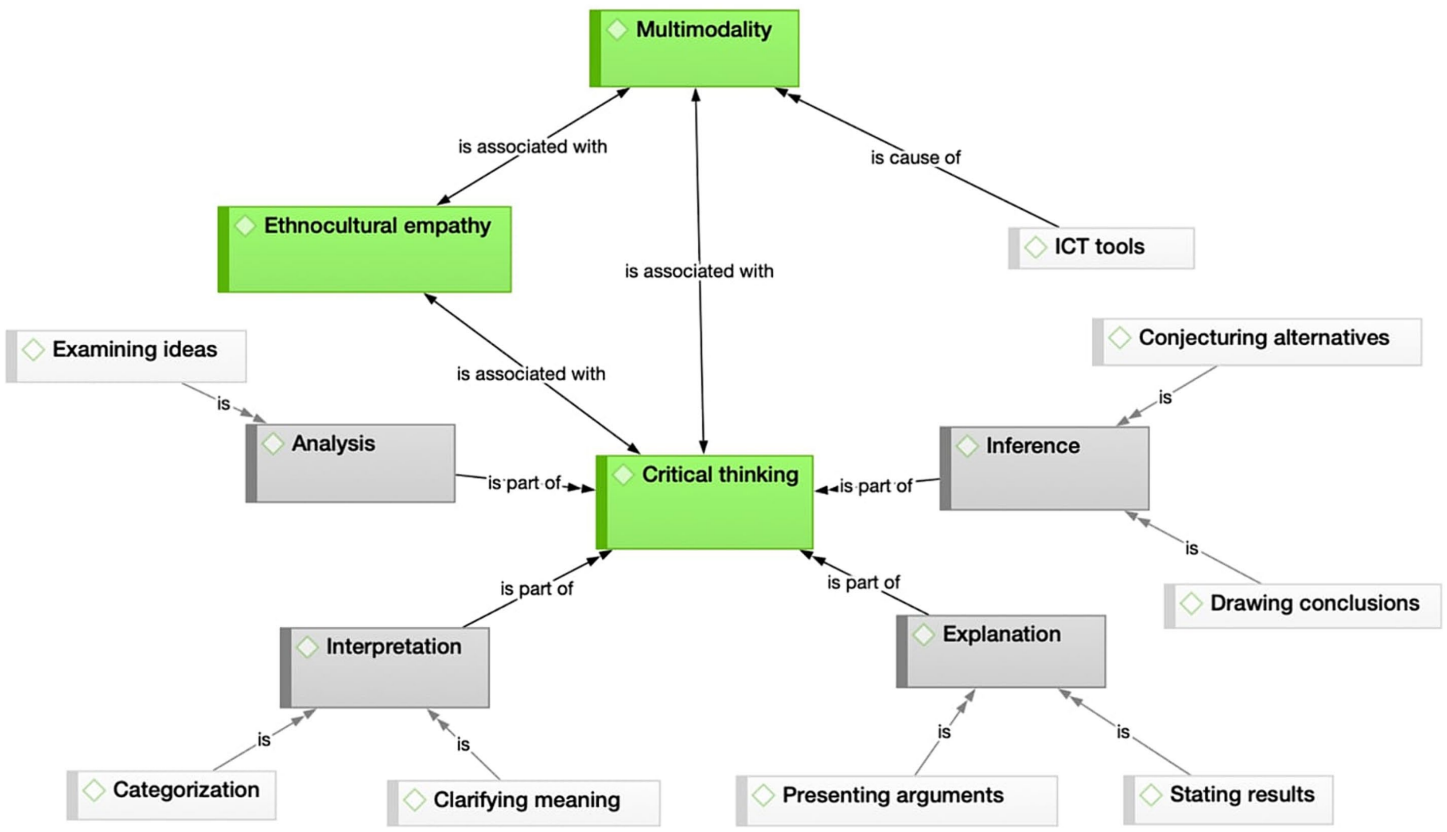
Relational patterns between the topics that emerge in the in-depth interviews (study 2).

In addition, a visual representation of the relationships between codes and categories for each subset was generated (Figures 1, 2) to enhance the understanding of data patterns. Color coding has been used to distinguish the main categories identified. Thus, the thematic nuclei that provide the backbone of this study, and the categories and subcategories described by Facione (1990, 2020), are shown in green, gray, and white, respectively. In Figure 1, in addition, the color red is added to round off Facione’s (1990, 2020) framework with the notion of metacognition, as reflected in the students’ logs.

Although the following section will detail the differences, similarities and implications of the inter-group comparison, a preliminary overview reveals a similar underlying structure, but also the greater complexity present in group 1, illustrated by the ‘self-regulation’ cluster in the top left of Figure 1.

This first visual approximation also provides a means of comparing the CT skills exercised by the two cohorts, which coincided in three of the four categories reported by each group, namely ‘inference’, ‘explanation’ and ‘interpretation’. Similarly, it is also possible to discern the absence in both groups of the category ‘evaluation’ that Facione’s (1990, 2020) framework includes.

A final consideration evidenced by the exploratory visual contrast between these two figures is that Figure 2 does not feature any allusions to teamwork, an aspect that will also be discussed in the sections below.

## 3 Results

### 3.1 Quantitative

Regarding the first research question, a first approximation is made based on quantitative data. Simple statistical analysis was used initially to describe the data that had been collected and variables that will be later analyzed. The factor scores for each of the factors in the RSEE were used to perform the subsequent analyses.

To descriptively compare the differences between before and after the intervention, the minimum and maximum scores registered (Mean = 0) are displayed in Table 3.

**Table 3:** Descriptive statistics for the total sample in each factor before and after the intervention.

Variable	*N*	Minimum	Maximum	Variable	Minimum	Maximum
PRE_Cultural openness and desire to learn	42	−3.71379	1.09919	POST_Cultural openness and desire to learn	−2.87672	1.06808
PRE_Awareness of contemporary racism	42	−2.73111	1.20204	POST_Awareness of contemporary racism	−2.24118	1.24376
PRE_Empathy	42	−2.64050	1.20254	POST_Empathy	−2.46930	1.34661
PRE_Resentment and cultural dominance	42	−1.40086	2.70647	POST_Resentment and cultural dominance	−1.37548	2.03130
PRE_Anxiety and lack of multicultural self-efficacy	42	−1.02781	2.42113	POST_Anxiety and lack of multicultural self-efficacy	−0.88682	3.30589

As we can see in Table 3, among the studied sample, the lowest scores before the intervention, as well as after the intervention, were obtained in Factor 1 (Cultural Openness). Their highest scores before the intervention were obtained in Factor 4 (Resentment) whilst after the intervention, the highest were obtained in Factor 5 (Anxiety). Overall, the results point in the desired direction. There is a slight improvement in all factors after the intervention, except for cultural openness. In addition, the results indicate a decrease in the lowest scores after the intervention; although they are still negative, in some cases they have improved by one point. However, the differences found before and after the intervention for the whole sample are not statistically significant.

**Figure 3:**
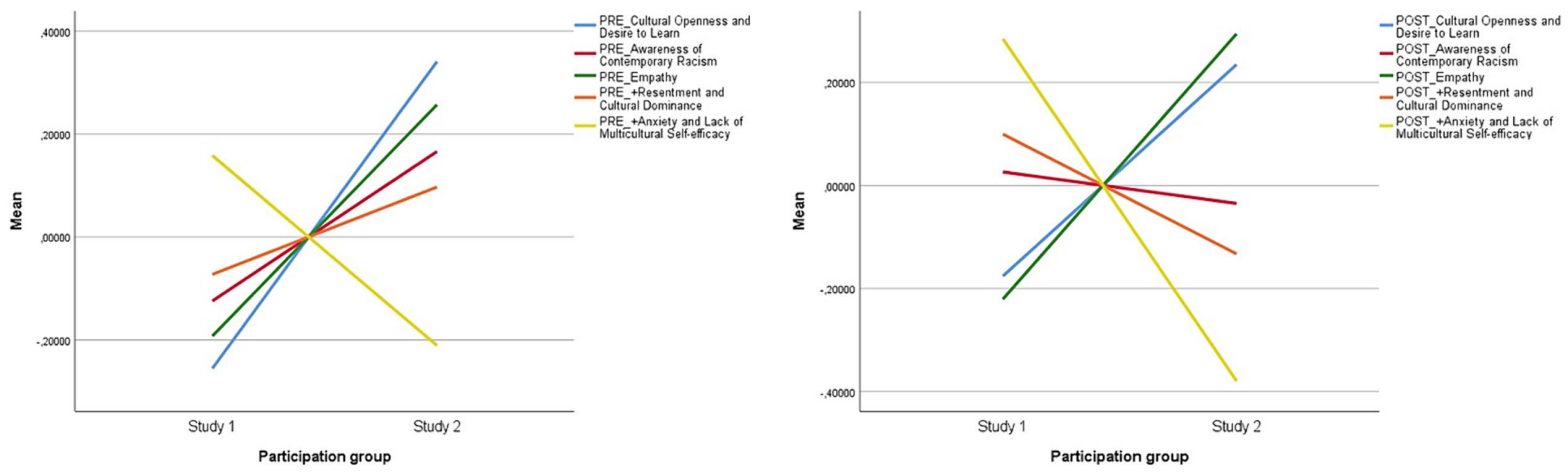
Changes between the two experimental groups before and after the intervention.

Moving on to the second research question, to know whether ethnocultural empathy unfolds differently depending on the type of multimodal product designed, we aimed to contrast the ethnocultural empathy levels of the two groups of participants who produced different final outcomes during the interventions: study 1 and study 2. Figure 3 shows how the factors differed between the two experimental groups before and after the intervention. From the figure we can observe that the results of study 1 for all factors except for Factor 3 (Empathy) show an increase after the intervention when contrasted with study 2, especially in Factor 2 (Awareness of Racism) and 4 (Resentment). That is, in study 1 there is an increase in Awareness, Anxiety and Resentment. On the contrary, study 2 shows a decrease in Factor 2 (Awareness of Racism), Factor 4 (Resentment) and Factor 5 (Anxiety). However, the changes within each group were not statistically significant.

Non-parametric tests were used (non-normal distribution was found in all factors of the scale) to compare the differences between the levels of ethnocultural empathy of the participants in the two studies. Table 4 shows the results obtained from the preliminary analysis, before and after the intervention.

**Table 4:** Test statistics for the Ethnocultural Empathy Scale factors and the participation group^a^.

		Cultural openness and desire to learn	Awareness of contemporary racism	Empathy	Resentment and cultural dominance	Anxiety and lack of multicultural self-efficacy
PRE	Mann–Whitney *U* test	127.5	189.5	148	203	160.5
	*Z*	−2.25	−0.674	−1.728	−0.33	−1.424
	Sig.	0.024	0.501	0.084	0.741	0.154
POST	Mann–Whitney *U* test	153.5	201	140	176.5	135.5
	*Z*	−1.589	−0.381	−1.932	−1.004	−2.071
	Sig.	0.112	0.703	0.053	0.315	0.038

^a^Grouping variables: participation group (Study 1 or study 2).

In Factor 3 (Empathy) and Factor 5 (Anxiety), significant differences were observed between the groups and their post-intervention results, with a *p* value of 0.053 and 0.038, respectively.

What stands out from Table 5 is the difference between both groups and their mean ranks within the previously mentioned factors post-moment. Interestingly, the results for Factor 3 (Empathy) were higher in study 2 (25.72 in study 2 and 18.33 in study 1), whilst the results for Factor 5 (Anxiety) were higher in study 1 (24.85 in study 1 and 17.03 in study 2).

**Table 5:** Test statistics for Factor 3 and Factor 5 of the Ethnocultural Empathy Scale and the participation group.

Course of participation		*N*	Mean rank	Sum of ranks
POST_Empathy	21–22	24	18.33	440.00
	22–23	18	25.72	463.00
	Total	42		
POST_Anxiety and lack of multicultural self-efficacy	21–22	24	24.85	596.50
	22–23	18	17.03	306.50
	Total	42		

The effect size r was calculated, obtaining a size effect of 0.625 for Factor 3 (Empathy) and 0.674 for Factor 5 (Anxiety). These results indicate that the significant differences and the size effect between both groups are large (Cohen, 1992, 2016).

Regarding research question 3, different variables were analyzed to determine their relationship with the development of ethnocultural empathy. No statistically significant results were obtained regarding academic performance or involvement in volunteering initiatives. Other socio-demographic variables, such as age or nationality and mother tongue, were discarded due to the homogeneity of the sample for the former and its excessive dispersion and reduced sample for the latter.

However, significant differences were found between the sexes. In this case, non-parametric tests were used (non-normal distribution was found in all factors of the scale). It is noticeable that Factor 3 (Empathy) also showed differences between the sexes (Table 6).

**Table 6:** Test statistics for the Ethnocultural Empathy Scale factors and sex of the participants^a^.

		Cultural openness and desire to learn	Awareness of contemporary racism	Empathy	Resentment and cultural dominance	Anxiety and lack of multicultural self-efficacy
PRE	Mann–Whitney *U* test	108.5	125	133	171	155.5
	*Z*	−2.178	−1.728	−1.51	−0.476	−0.906
	Sig.	0.029	0.084	0.131	0.634	0.365
POST	Mann–Whitney *U* test	141.5	126	109	147	167
	*Z*	−1.279	−1.7	−2.163	−1.13	−0.592
	Sig.	0.201	0.089	0.031	0.259	0.554

^a^Grouping variables: Sex.

According to the mean ranks in Table 7 for the mentioned factor, empathy levels in females (24.24) were higher than empathy levels in males (15.38).

**Table 7:** Test statistics for Factor 3 and Factor 5 of the Ethnocultural Empathy Scale and the sex of the participants.

Sex		*N*	Mean rank	Sum of ranks
POST_Cultural openness and desire to learn	Male	13	17.88	232.50
	Female	29	23.12	670.50
	Total	42		
POST_Awareness of contemporary racism	Male	13	16.69	217.00
	Female	29	23.66	686.00
	Total	42		
POST_Empathy	Male	13	15.38	200.00
	Female	29	24.24	703.00
	Total	42		
POST_Resentment and cultural dominance	Male	13	24.69	321.00
	Female	29	20.07	582.00
	Total	42		
POST_Anxiety and lack of multicultural self-efficacy	Male	13	23.15	301.00
	Female	29	20.76	602.00
	Total	42		

Due to the small population sample, effect size r analysis was calculated to check the relevance of the differences. A size effect of 0.708 for Factor 3 (Empathy) was obtained, indicating that the significant differences and the effect size between both groups are large (Cohen, 1992, 2016).

Finally, it was sought to establish whether there is a statistical correlation between empathy and CT skills. Thus, the correlations between the CT analysis and factors based on Facione (1990, 2020) and the five factors of the RSEE were tested using non-parametric correlations (non-normal distribution was found in all factors of the scale). As observed in Table 8, no significant differences were found, and, in any case, a negative, small correlation was found between them.

**Table 8:** Correlations between the critical thinking analysis and factors based of Facione and the five factors of the Empathy Ethnocultural Scale.

			Critical thinking factors (Facione)	POST_Cultural openness and desire to learn	POST_Awareness of contemporary racism	POST_Empathy	POST_ + Resentment and cultural dominance	POST_ + Anxiety and lack of multicultural self-efficacy
Spearman’s Rho	Critical thinking factors (Facione)	Correlation coefficient	1.000	−0.034	−0.174	−0.119	−0.162	−0.017
		Sig. (two-tailed)		0.875	0.417	0.578	0.449	0.938
		*N*	24	24	24	24	24	24

### 3.2 Qualitative

As this is a mixed-methods study, qualitative data was collected and analyzed to get a more accurate picture and a deeper insight into the affective and cognitive processes that the participants went through. They will also enable us to answer the research questions 4 and 5.

Thus, the narratives contained in the students’ diaries, which were written entirely in English, and the accounts expressed during the interviews, conducted in Spanish and translated by the authors, complement the results contained in the statistical analysis above. The theoretically grounded categories and the most recurrent quotes from the respondents frame the structure of the presentation of the findings in this section. For clarity, this section will report the results of each of the cohorts independently hence structured in two separate sub-sections.

#### 3.2.1 Study 1: teaching materials for low-literacy migrant adults

Regarding the first study cohort, where students were arranged into seven work teams, participants’ logs reflect on their experiences during the collaborative, multimodal intervention, and how they relate to the development of their empathetic attitudes and their deployment of critical thinking skills.

##### 3.2.1.1 Empathy

Empathy is largely ascribed to emotional states prompted by the realization of the linguistic and cultural hurdles that migrant students must overcome. This overlapping of personal and third-party affective states results in feelings of dismay in the face of adversity (S2), admiration towards the resilience displayed by migrants (S12) and a firm conviction that “emotional factors need to be taken into account when developing a [SL teaching] project” (S22).

One outcome of this empathic development is the enhancement of competences in the crafting of instructional SL materials, spurred by the acknowledgement that they contribute to the successful inclusion of low-literacy migrant adults.

Finally, an increased “awareness of the situation and problems of this collective” (S26) is also cited as being conducive to a higher level of empathy.

##### 3.2.1.2 Critical thinking skills

The salience of the interplay between critical thinking and the adoption of a multimodal approach in the production of the final products is manifest. As S13 relates:

I have learnt of the importance of employing multimodal materials for teaching and acquiring a successful learning in students. In that sense, for the transmission of content it is essential to work with the visual, audio, gestural, linguistic, and spatial designs.

This reflective process upon completion of the intervention spans the suitability of the materials produced for the Spanish language learners for whom they are intended. For instance, S20 remarks on “being able to choose the right activities for each language level and being able to judge if an activity is good enough or appropriate for the type of learner we had.”

In turn, the logs reflect, as expected, a remarkable level of metacognition, operationalized as the analysis of the self-process of learning, the successful self-directed learning strategies adopted, as well as assessments of what favors or hinders their acquisition of skills or competencies.

##### 3.2.1.3 Multimodality

The relevance of multimodality to better respond to the needs of the target audience of these materials is consistently referred to, as a result of which feelings of empathy and commiseration with the hardships experienced by migrants – especially those with languages and cultures of origin distant from the majority in Spain, or those with an incipient level of prior training in languages other than their mother tongue – are expressed.

Moreover, multimodality is widely associated with ‘diversity’ and ‘interculturality,’ which further enhances the plasticity of this concept to accommodate the possible heterogeneity of future learners’ needs. Multimodality thus stands as a resource for navigating uncertainty and enhancing the adaptability of the trainee teacher.

##### 3.2.1.4 Teamwork

The process of reflection and monitoring of one’s own progress is expressed fundamentally through the contributions of one’s own individual work to the group effort. In this regard, S3 reflects:

Working in a team is ideal for learning about the way colleagues work and the ideas that each of them can contribute, the attitude is much more positive and makes the task more interactive. It also helps to foster the creativity of each team member.

In the same vein, group work is associated with concepts such as ‘motivation,’ ‘brainstorming’ and ‘enriching’ practices, both from an affective and a cognitive perspective. Thus, S19 comments on her sense of fulfillment by saying: “I took many ideas from my classmates, reformulated some others and had the feeling of being listened to regarding my own ideas.”

Similarly, the benefits that the collaborative approach can unlock for soft skills are identified: “I think that working in a group is always beneficial as you get to know other points of view and work on adaptation and mediation” (S17).

In contrast, the collective approach is not unanimously considered in the records analyzed. The preference for individual work is rooted in factors such as time management and productivity (S2), workload allocation (S7), coordination (S8, S21, and S24) or the reconciliation of conflicting ideas (S22), which would be undermined by group work.

#### 3.2.2 Study 2: video about three low-literacy adult migrants

As for the second group, during the in-depth interviews, students verbalized their reflections, attesting to their critical thinking skills. These, in turn, shed light on the ways in which digitally-based multimodal language teaching is deemed to better meet the needs of migrant learners.

##### 3.2.2.1 Empathy

The most prominent component of empathy in this group is cognitive in nature, taking the form of being able to understand or explain something to somebody who is culturally distant. Moreover, for this approach, empathy is perceived as a two-way street, as empathizing more with immigrants will “make it possible for them to understand us too” (S28).

##### 3.2.2.2 Critical thinking skills

Interviewees’ critical thinking encompasses, among others, making inferences. As S29 elaborates:

In a primary school it would be very complicated to have to explain bureaucracy to [migrant] children. But in secondary school and perhaps beyond it would be much simpler, although it would still be a slightly more complex task than explaining it to an adult. But maybe some workshops could be held in which they could be given an explanation.

The video-based intervention also enabled participants to hone their skills in making interpretations based on the information available, as exemplified by S28 stating that:

It would have been the same [if] I had had to go to Germany when I was a child, I suppose, and possibly my classmates would have ignored me and not helped me, it would have upset me, because what could I have done without having someone to lend me a helping hand? So I think that teachers themselves should raise awareness among their students.

Similarly, the ability to provide explanations is evident in interview excerpts, such as this one, about the interplay between multimodal and traditional approaches to teaching:

I believe that joining forces goes a long way, because if you are constantly reading one thing or you are always doing the same thing, even if it is audiovisual, in the end, it is going to end up being monotonous. But if there is a change [...] a perfect balance can be achieved and it becomes, firstly, more impactful for the student [...], and secondly, it makes it much more memorable [...]. So I think the right compromise between both types of education is the right approach that will lead to much more progress for the learner. (S29)

Finally, in contrast to the participants in group 1, group 2 interviewees exhibited analytical CT, embodied in the examining of the ideas of their peers. The following is the S28’s reaction to S29’s remark above:

I share your view because I believe that a variety of approaches is what brings about the right outcome, since doing the same thing on a daily basis, as you have pointed out, eventually becomes monotonous.

##### 3.2.2.3 Multimodality

Multimodality, enabled by digital tools, is believed to be instrumental in cultivating competencies that could not be otherwise acquired by the intended recipients of the learning materials:

In other words, when you ask a student to write a text commentary, you are assessing their ability to express themselves, to reason... […] and submit them in writing. However, the moment you screen a video, perhaps so that they can elicit information, other types of skills come into play, such as being able to interpret what they are viewing and how to attribute meaning to it. (S30)

In this regard, as Peña-Acuña and Cislowska (2023) point out, multimodal resources enable multilevel learning, where learners incorporate knowledge about a particular digital tool alongside the appreciation of how such tool “changes the way of interacting among equals and with the world and for what other purposes the learned tools can be useful” (p. 278).

#### 3.2.3 Final products

As for the end products participants designed in study 1, all seven work teams identified topics of interest for the target population, such as, shopping at the supermarket, buying food, daily routines, applying for a job, and moving around the city; except for one group that suggested creating a blog about cities to visit -a regular theme in mainstream textbooks. Full integration of multimodal elements was achieved to varying degrees, apart from written language elements, three of the seven teams only included simple visual support; in other cases, visual aids were used to facilitate comprehension and enhance the language learning process. Four groups included digital resources and suggested the use of digital technologies in the learning process (*Kahoot*, *Tiktok* and mobile phones); three groups added audiovisual elements and used digital-based apps to create the materials; finally, one group recorded their own audio files for the teaching unit. In general, although many of the groups designed attractive and thoughtful, and generally well sequenced tasks, in most of the cases activities were too difficult for the low-literacy adult learners they were intended for.

It is noteworthy that two groups included a third language, that of the target population they had selected, which in both cases was Ukrainian. This choice is in line with the findings of Ávila-López and Rubio-Alcalá (2023), who claim that, to fully empower individuals, “multimodal modes should definitely include the linguistic repertoires that migrants bring to the social equation” (p. 182).

Turning to study 2, student teachers were asked to produce a video containing the main ideas of an article about the acculturation process of migrants. As explained above, participants were required to transform the written article in a digital multimodal product, a video file with several elements: they had to add images, subtitles, and their own voices to narrate the contents; and at the end of the video, three comprehension questions would help viewers to check what they had learnt watching the video. Half of the groups managed to design and create elaborated videos that fulfill, to varying degrees, all of the criteria. Two of the groups performed even beyond expectations. In general, students identified key concepts and defined them using their own words, adapted contents and language to make ideas more accessible, selected a wide variety of elements to support and illustrate their ideas, added subtitles and their own voices, paying special attention to intonation in order to attract the audience’s attention. The rest of the groups accomplished the task moderately well, including most of the elements required. Lower attainment was due to limited variety of digital elements and in 2 cases, also a lack of cognitive effort as participants mainly summarized the contents of the reading. In most of the groups there is, however, an important display of cognitive abilities and CT skills. Student teachers showed a good understanding and analysis of the text, identifying key information; they managed to draw relevant conclusions from the reading and presented their ideas in the video supporting them with sound arguments and illustrating them with multimodal elements. Thus, the text mediation competences, as described in the Companion Volume of the Common European Framework of Reference for Languages (Council of Europe, 2020), were extensively demonstrated in the final products. There is a clear effort on the part of the participants to make high-quality attractive videos.

## 4 Discussion

As stated at the beginning of this work, this study set out to compare how effective two digital multiliteracy interventions were in developing future language teachers’ ethnocultural empathy and cognitive abilities when appraising the educational needs of low-literacy migrants. The analysis of the quantitative and qualitative data has provided answers to the research questions posed at the beginning of the study, which aimed to assess the potential for developing the ethnocultural empathy of student teachers via the design of multimodal products.

Data has shown that student teachers’ ethnocultural empathy can be developed by introducing multimodal tasks in their training courses, which is in line with Rasoal et al.’s (2011a) assertions that ethnoculturally empathic stances are amenable to learning and training. However, it seems that the nature of the final products created by participants, leaning more towards either affective or cognitive perspective-taking (Jiang and Gao, 2020) in each of the two groups featured in this work, has significantly impacted the extent of such development.

This allows us to address the second research question, quantitatively, it has been possible to establish significant differences between groups after the interventions, with a sizable effect. Such differences have been found in Factor 3 (Empathy) and Factor 5 (Anxiety), together with the high effect sizes obtained. This strongly implies that the type of multimodal product that participants are invited to create significantly impacts on the development of ethnocultural empathy and that these results can be generalized. Accordingly, depending on the type of final product expected of them and the type of evaluation they anticipated would be applied, participants would potentially develop greater or lesser anxiety and empathy levels after the intervention. Hence, we have ascertained that the group participating in study 2 showed higher levels of empathy and the participants of study1, lower levels of anxiety according to the factors obtained in the Revised Ethnocultural Empathy Scale.

With regards to the third research question, another significant finding of this study is the gender differences revealed. Quantitative results show higher levels of empathy in females, which is consistent with previous studies (Cundiff and Komarraju, 2008; Rasoal et al., 2011b; Pinzón Corredor, 2018). It is, however, worth noting that the large size effect counterbalances the small population sample size in terms of generalizability. No other demographic variables analyzed, however, produced statistically significant results.

The quantitative data have not provided the anticipated results, not relating participants’ cognitive and affective empathy levels to one another in a significant manner. This may be due to the fact that in the Spanish version of the RSEE, cognitive and affective empathy are merged into one single factor, possibly rendering its distribution of factors inadequate for a population of prospective L2 teachers of migrant learners. It seems necessary to verify whether the tool operates well with such a population, hence a study with a larger sample is needed. Thus, the small sample size is insufficient to draw robust comparable results. However, the participants’ contributions are rich in insights and nuances at the qualitative level, which allows us to answer research questions 4 and 5.

As to the fourth research question, pertaining to the cognitive processes/capabilities and CT skills that are promoted when designing multimodal products intended for adult immigrants, the findings presented in this paper are consistent with the conclusions of Peña-Acuña and Navarro-Martínez (2024), whose study on the training of student teachers in the use of multimodal storytelling applications found an increase in trainees’ mental flexibility (p. 15). In addition, the adoption of the multimodal approach seems to have elicited a considerable degree of metacognition in the students. Despite not being statistically supported, it is an interesting finding, since it might point to the need to extend Facione’s (1990, 2020) CT framework. Along the same lines, Dwyer et al. (2014) integrative framework articulates this metacognitive capacity in terms of ‘reflective judgment’ and ‘dispositional factors’, both of which “ultimately dictate how well each thinking process will be carried out” (p. 49).

As for the fifth research question, the data collected in the reflection logs and interviews sheds light on the participants’ perceptions gained from their experience during the production of the multimodal outputs. In terms of cognitive processes and CT skills, qualitative data suggest a diverging activation of CT skills dependent on the required multimodal task. Some inter-group differences have been observed, as in the view of the participants in group 1, the interventions carried out enabled them to deploy their CT skills and to hone their competencies to work in teams or in collaboration with their peers in the pursuit of a common goal. This echoes previous studies linking cognitive and action-oriented tasks (Dwyer et al., 2014), where learners are expected to jointly deliver a multimodal final product that also relies on mediation and problem-solving skills. In group 2, however, the participants showed a more analytical approach to working collaboratively, engaging their CT skills to offer feedback to the arguments held by their peers. While empathy and cognitive perspective-taking are both valuable in promoting understanding and collaboration among diverse groups, their exclusive focus may overlook the complexity of emotional responses and interpersonal dynamics in intergroup relations.

As for the relationship between ethnocultural empathy and CT skills in prospective teachers of Spanish as an L2, this study has provided valuable qualitative insights into the role that digital technology-based multimodal interventions have the potential to fulfill. Thus, in the first group, responsible for the production of instructional materials, the participants exhibited a higher level of complexity in their CT skills, especially in the area of self-regulation. This group also demonstrated effective learning strategies that facilitated, to varying degrees, successful task finalization and the cultivation of ethnocultural empathy, underscoring the importance of empathy in the design of learning materials. Along these lines, ethnocultural empathy seems to reconcile cognitive and affective elements, where knowledge and understanding of the challenges faced by migrants are added to the emotional triggers through which empathetic stance-taking arises. However, some difficulties have been identified. Although integrating multimodal elements appears to be manageable for most of the groups, the requirement to consider the entry level of the prospective audience has been the most challenging element for the trainee teachers. That is, although they were more conscious of migrants’ needs, not all of them succeeded in transferring that knowledge into a fairly novel task for them. In line with Zhang-Leimbigler’s (2014) remarks, the multiliteracies approach fostered participants’ observing and analyzing skills, but maybe the task was beyond their zone of proximal development (Macy, 2016), hindering the transferability of knowledge. Misalignments in the level of competence such as this may happen when applying new pedagogical theories in teacher education courses. In fact, as some participants reported, this type of tasks may require more time, or the inclusion of facilitating preparatory tasks. They would have also liked to “put the activities into practice with migrants” (S8) in order to check their effectiveness.

The high level of self-awareness, as shown by constant references to self-regulation skills, together with the probably overwhelming novelty of the assignment could also explain the higher levels of anxiety pre-post in study 1. Prioritizing perspective-taking towards oneself, as Wallace-Hadrill and Kamboj (2016) recall, may attenuate positive emotions. This explains how the intervention in study 1 made future teachers fully aware of their detachment from the situation and needs of migrants, which in turn led them to doubt their multicultural self-efficacy and experience higher levels of anxiety.

The second group, engaged in the creation of a video presentation, demonstrated their CT skills and acknowledgement of the advantages of a multimodal digital approach to language teaching for migrant learners during the in-depth interviews. Their reflections drew attention to the benefits of incorporating a variety of modalities in the creation of educational content to better serve the needs of low-literate adult migrants. Similarly to what Wang et al. (2014) report, in this group empathy is operationalized primarily as cognitive understanding of culturally distant individuals, facilitating both mutual appreciation and reciprocal consideration. However, the absence of the category ‘evaluation’ in their CT skills as described in Facione’s (1990, 2020) framework suggests areas of further development in terms of their metacognitive processing and self-regulation. Unlike participants in study 1, these learners seem to be faced with a more manageable task that allows them to activate their CT skills and, even more so, to make the most of their digital abilities. The introduction of audiovisual multimodal elements appears to have facilitated the successful completion of the task. On the other hand, the higher levels of empathy and lower levels of anxiety in study 2 show that this multiliteracies design significantly improves the ethno-cultural empathy of the prospective teachers of migrant pupils; as they are more familiar with the reality of the target population and feel more capable of carrying out the required task, their anxiety and lack of multicultural self-efficacy levels decrease.

In general, although not supported by quantitative data, participants’ narratives indicate a change in their perspectives and beliefs about migrants and their own future jobs. Trainees verbalize their awareness-raising process ‘before this I was not so aware of the difficulty migrants have because of not knowing the language of the country they are arriving in’ (S8). Besides, many of them admitted to being motivated by working on tasks that could be useful for their future work. There was also a sense of pride in the effort they had put in and the product they had created.

We agree with Gouthro and Holloway (2013) when they affirm that in teacher education “we need to be committed to strategies to carefully prepare and support teachers to use both critical lifelong learning and multiliteracies approaches in their own teaching practices” (p. 57). Multiliteracies practices in technology-mediated settings enhance teacher education, as they help teacher students become critically reflective educators who can prepare their own learners for the challenges of an ever more plurilingual multicultural and digital society. Our findings also corroborate the claims of Warner and Dupuy (2018) about future language teachers having fixed conceptions of language learning and teaching, and when confronted with situations that do not reflect their experiences and ideas tensions arise. As suggested by the authors, the interventions presented here enhanced students’ reflection, which is necessary to go through the transformation process specific to the multiliteracies framework. In this way, this study contributes to filling the gap identified by Paesani and Allen (2020) when they advocate for language teacher programs that enhance the understanding of the multiliteracies framework. Thus, the findings in this study support the value of integrating digital media, CT skills and ethnocultural empathy development into teacher education programs to equip future language teachers with the skills and awareness necessary to function effectively in diverse educational settings and meet the needs of multicultural learner populations.

The authors acknowledge the limitations present in this work, mainly in three respects. Firstly, given that the instrument used to collect quantitative data is the RSEE, which has been calibrated on the basis of a scale validated in a Colombian sample but not in a Spanish one, and in view of the low statistical significance observed in most of the factors examined in this study, a broader population survey would be required in order to check its validity. Secondly, the uneven ratio of female and male subjects may have influenced the results obtained. Thus, a more representative sampling of men and women in the study group might refine the reported figures. Finally, the reluctance of students to participate in qualitative research, especially their unwillingness to respond to in-depth interviews, casts doubt on the generalizability of the data obtained. Follow-up studies would be necessary to confirm the reported results.

Despite the small sample size, the study certainly adds to our understanding of the impact of multimodal tasks involving critical thinking skills on trainees’ cognitive and affective abilities. Besides, it adds to the growing body of research that emphasizes the desirability of embedding multimodal digitally-based content creation tasks in training curricula for future language teachers. Those multiliteracy tasks appear to enhance future teachers’ ethnocultural empathy, particularly among females, reduce their anxiety and perceived lack of multicultural self-efficacy, and foster the deployment of learners’ CT skills, as well as their awareness of and interest in their future teaching practice.

## Data availability statement

The raw data supporting the conclusions of this article will be made upon request by the authors, without undue reservation.

## Ethics statement

Ethical review and approval was not required for the study on human participants in accordance with the local legislation and institutional requirements. The studies were conducted in accordance with the local legislation and institutional requirements. The participants provided their written informed consent to participate in this study.

## Author contributions

AF-C: Formal analysis, Writing – review & editing, Writing – original draft, Supervision, Resources, Project administration, Methodology, Investigation, Conceptualization, Funding acquisition. EC-B: Writing – review & editing, Writing – original draft, Supervision, Methodology, Investigation, Formal analysis, Data curation. PF-A: Writing – review & editing, Writing – original draft, Methodology, Formal analysis, Data curation.
