# Corrigendum: Ethnocultural empathy development of future language teachers through digital multiliteracy resources for low-literacy adult migrants

**DOI:** 10.3389/fpsyg.2024.1462241

**Published:** 2024-07-19

**Authors:** Analí Fernández-Corbacho, Esther Cores-Bilbao, Patricia Flor-Arasil

**Affiliations:** ^1^English Studies Department, Universidad de Huelva, Huelva, Spain; ^2^Faculty of Humanities and Social Sciences, Universidad Isabel I, Burgos, Spain; ^3^Faculty of Health Sciences, Universidad Internacional de Valencia, Valencia, Spain

**Keywords:** ethnocultural empathy, language teacher education, critical thinking skills, multiliteracy, digital resources, multimodality

In the published article, there was an error in the Funding statement. Information from one of the sources of funding (COIDESO Research Center) was missing. The text that appeared in the original version was as follows: The author(s) declare that financial support was received for the research, authorship, and/or publication of this article. This study is a result of the RDI Project Multiliteracies for adult at-risk learners of additional languages (MultiLits), REF. PID2020-113460RB-I00 funded by MCIN/AEI/10.13039/501100011033.

The correct Funding statement appears below.

